# Compound heterozygous loss of function variants in *MYL9* in a child with megacystis–microcolon–intestinal hypoperistalsis syndrome

**DOI:** 10.1002/mgg3.1516

**Published:** 2020-10-08

**Authors:** Justin L. Kandler, Evgenia Sklirou, Audrey Woerner, Leslie Walsh, Eleina Cox, Yuan Xue

**Affiliations:** ^1^ Fulgent Genetics Atlanta Georgia USA; ^2^ Division of Medical Genetics Department of Pediatrics Children's Hospital of Pittsburgh Pittsburgh Pennsylvania USA

**Keywords:** congenital, loss‐of‐function, MMIHS, *MYL9*, myopathy

## Abstract

Megacystis–microcolon–intestinal hypoperistalsis syndrome (MMIHS), or “visceral myopathy,” is a severe early onset disorder characterized by impaired muscle contractility in the bladder and intestines. Five genes are linked to MMIHS: primarily *ACTG2*, but also *LMOD1*, *MYH11*, *MYLK*, and *MYL9*. Here we describe a three‐year‐old girl with bilateral hydronephrosis diagnosed at 20 weeks gestation and congenital mydriasis (both of which have been previously observed among individuals with MMIHS). A clinical diagnosis of MMIHS was made based upon the presence of megacystis, lack of urinary bladder peristalsis, and intestinal pseudo‐obstruction. After initial testing of *ACTG2* was negative, further sequencing and deletion/duplication testing was performed on the *LMOD1*, *MYH11*,*MYLK*, and *MYL9* genes. We identified two heterozygous loss of function variants in *MYL9*: an exon 4 deletion and a nine base pair deletion that removes the canonical splicing donor site at exon 2 (NM_006097.5:c.184+2_184+10del). Parental testing confirmed these variants to be *in trans* in our proband. To our knowledge, only one other individual with MMIHS has biallelic mutations in *MYL9* (a homozygous deletion encompassing exon 4). We suggest *MYL9* be targeted on genetic testing panels for MMIHS, smooth muscle myopathies, and cardiovascular phenotypes.

## Graphical abstract

In this manuscript, we describe a proband with a clinical diagnosis of congenital MMIHS and two rare compound heterozygous loss of function variants in MYL9, which we confirmed to be in trans by parental testing. To our knowledge, this is only the second peer‐reviewed report of a genetically characterized case of MYL9‐associated MMIHS and supports the gene‐disease association between MYL9 and MMIHS with an autosomal recessive inheritance pattern. We conclude that MYL9 be targeted on genetic testing panels for MMIHS, smooth muscle myopathies, and cardiovascular phenotypes.

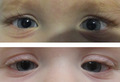

## 1 INTRODUCTION

The most well‐studied gene associated with MMIHS is *ACTG2* (OMIM: 102545) (Thorson et al., 2014; Wangler et al., 2014), which has autosomal dominant inheritance. To date, four other genes are also linked to MMIHS among children born to consanguineous parents, suggesting autosomal recessive inheritance: *LMOD1* (OMIM: 602715), *MYH11* (OMIM: 160745), *MYLK* (OMIM: 600922), and *MYL9* (OMIM: 609905) (Halim, et al., 2017; Moreno et al., 2018; Thorson et al., 2014; Wangler et al., 2014). All five genes are involved in actin/myosin II activity (Moreno et al., 2018; Sanders et al., 2012), explaining their impact on smooth muscle contractility. Most recently, loss of function variants in *MYL9* was reported in one individual with MMIHS whose sibling died of renal failure associated with suspected MMIHS at one month of age [evaluation of variants in the sibling was not possible due to DNA degradation; (Moreno et al., 2016, 2018)].

*MYL9* [previously referred to as “MLC‐2”; (Kumar et al., 1989)] is an 8.3 kb gene located on chromosome 20 (GRCh37:g.35169887_35178226; GRCh38:g.36541519_36551447) that encodes the 20 kDa myosin regulatory light chain 9 (MYL9), previously known as “MLC20” [https://www.ncbi.nlm.nih.gov/gene/10398; (Moreno et al., 2018; Sanders et al., 2012)]. Two different transcripts are reported in human expression studies from the GTExPortal (https://gtexportal.org/home/gene/MYL9#geneExpression): NM_000697.5 (ENST00000279022.7) and NM_181526.3 (ENST00000346786.2). The first and more highly expressed transcript, NM_000697.5, has four exons (one non‐coding) and encodes a protein of 172 amino acids in length, while the second transcript, NM_181526.3, has three exons (one non‐coding) and encodes a protein 118 amino acids in length due to omission of exon 3. Generally, *MYL9* mRNA is most prevalent in cells comprising arterial, bladder, colon, and esophageal tissues, with lower but still above baseline expression in cervical, fallopian tube, prostate, and uterine tissues (Figure S1). As might be expected for a gene connected with only smooth muscle myopathies, *MYL9* mRNA expression is quite low in skeletal and cardiac muscle.

Phosphorylation of MYL9 occurs at p.Ser19, stimulates myosin II ATPase activity, and subsequently results in cross‐bridge cycling and contraction; dephosphorylation of MYL9 leads to relaxation [reviewed in (Li et al., 2018)]. The authors of the first *MYL9* clinical report suggested that loss of *MYL9* due to homozygous deletion of exon 4 disrupts normal MYL9 expression and possibly compromises actin‐myosin binding, ultimately diminishing the ability of smooth muscle cells to contract (Moreno et al., 2018). Whether the observed phenotype was due to impaired phosphorylation of MYL9, impaired binding of MYL9 to myosin heavy chain II, or degradation of MYL9 due to severe truncation remains to be tested.

Here, we describe a proband diagnosed with MMIHS after previous observations of bilateral hydronephrosis, congenital mydriasis, megacystis, lack of urinary bladder peristalsis, and intestinal pseudo‐obstruction. Genetic testing of this individual and her parents revealed two compound heterozygous loss of function variants in the *MYL9* gene, consistent with the previous report of MMIHS associated with biallelic *MYL9* loss of function variants in another individual (Moreno et al., 2016, 2018).

## 2 MATERIALS AND METHODS

### 2.1 Editorial policies and ethical considerations

Patients were evaluated by clinical geneticists. Consent for the publication of photographs and clinical data was obtained from the proband's parents.

Initially, sequencing and deletion/duplication analysis were performed for the *ACTG2* (GenBank: NG_034140.1) gene in the proband by standard Illumina NGS at Prevention Genetics, which returned as negative. Subsequently, sequencing and deletion/duplication analysis were performed using extracted DNA from patient's and parents' blood samples on the *LMOD1* (RefSeq: NC_000001.11, nucleotides 201896456‐201946548, complement), *MYH11* (GenBank: NG_009299.1), *MYLK* (GenBank: NG_029111.1), and *MYL9* (RefSeq: NC_000020.11, nucleotides 36541519‐36551447) genes by standard Illumina NGS at Fulgent Genetics. Variants in *MYL9* were confirmed by standard orthogonal methods (Sanger for the intronic deletion, qPCR for the exon 4 deletion) and have been submitted to the ClinVar database (https://www.ncbi.nlm.nih.gov/clinvar/) with accession numbers VCV000818204.1 and VCV000818205.1. Primer sequences for the qPCR reactions: exon 4 coding region (forward 5′‐TGTATGTCTCAGCCCAAGTTC‐3′, reverse 5′‐TCTCGTCCACTTCCTCATCT‐3′) and the 3′UTR (forward 5′ATGGGAGTGTGCTCAGGA‐3′, reverse 5′‐CACAGACACACAGACCAGAAG‐3′). Alamut splicing predictors SpliceSiteFinder‐like, MaxEntScan, NNSPLICE, GeneSplicer, and Human Splicing Finder were queried to estimate the impact of the NM_006097.5:c.184+2_184+10del variant on splicing.

## 3 RESULTS

The proband, born in 2017, is a girl of Scottish, Irish, and German descent with MMIHS. She has three healthy older siblings (one sister and two brothers). Notably, her mother has a history of miscarriage of a male fetus at 20 weeks, and autopsy results showed posterior urethral valve anomaly and enlarged bladder possibly due to MMIHS or another disorder. The proband's paternal familial history is notable for the childhood deaths of great aunts at ages <1 and 12 year(s), respectively, due to unspecified heart problems. The proband's father is healthy, and her paternal grandfather is alive at 66 with no known heart issues. The proband's maternal familial history is notable for the death of her grandfather in his 50 s due to amyotrophic lateral sclerosis, and chronic constipation in her mother (bowel movement once/week), resolved by gastric bypass surgery in her 30 s.

The proband was born prematurely at 33 weeks, six days by C‐section. Fetal hydronephrosis and oligohydramnios were identified at 20 weeks gestation. To address this, fetal surgery was performed to place vesicoamniotic shunts, but was ineffective. Abdominal distention was present at birth, and X‐rays of her abdomen demonstrated the obstruction of the duodenum. She underwent surgery to release observed Ladd's bands, at which time she was diagnosed with megacystis with thickened wall and hypoperistalsis. These observations indicated possible MMIHS. She also underwent exploratory laparotomy, gastric tube placement, and broviac catheter insertion immediately post‐birth; Broviac replacement at five months; adhesiolysis, open gastrojejunostomy, and wedge biopsy of liver (tortuous duodenum noted) at six months; Broviac replacement at 13 months.

**FIGURE 1:**
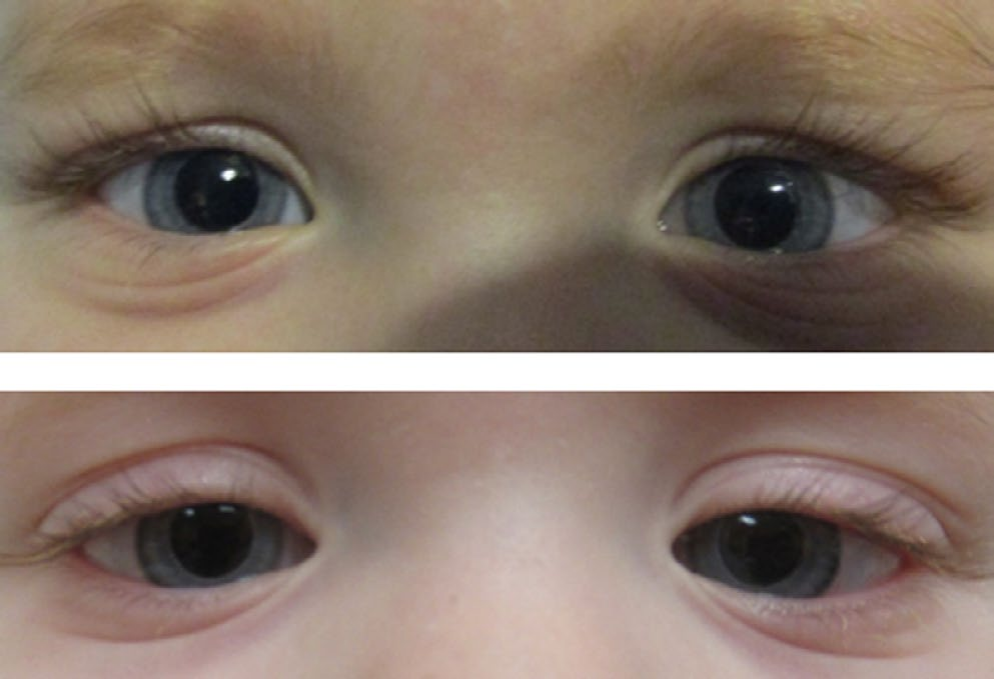
Congenital bilateral mydriasis in the proband. Photographs of the proband's eyes were taken at 19 months and 25 months of age (top and bottom, respectively). Note the reflection of the ceiling lights in the proband's pupils. Measurements of pupillary diameter were approximately six mm in both dark and light environments.

Other notable symptoms present in our proband and the other patients with MMIHS linked to *MYL9* are listed in Table 1. Importantly, our proband had congenital bilateral mydriasis (Figure 1), an immediately evident symptom that was also present in the other individual with *MYL9*‐associated MMIHS (Moreno et al., 2016, 2018). The proband continues to require total parenteral nutrition and has had multiple urinary tract infections requiring antibiotic prophylaxis and two respiratory infections. Additionally, she was hospitalized for rhinovirus infection and cyanotic episodes associated with respiratory syncytial virus infection in the first three months of life, as well as for an episode of respiratory distress at five months. A small bowel anatomical obstruction required hospitalization in the pediatric intensive care unit at 15 months, addressed surgically. The proband is currently enlisted (stage II) for small bowel transplant. Occasional kidney stones beginning at one year of age have required surgical removal and nephrostomy tube placement. Additionally, the proband suffers from progressive white matter loss of unknown etiology with lingual manifestations.

**TABLE 1:** Biallelic *MYL9* ^a^ variants among individuals with MMIHS

Case	Reference	Genotype^1^	Detailed phenotypes^3^
P1^2^ (deceased)	Moreno et al. (2016; Moreno et al. (2018)	DNA degradation, N/A	See Moreno et al. (2016), Moreno et al. (2018)
P2^2^ (deceased)	Moreno et al. (2016), Moreno et al. (2018)	[6964 bp homozygous deletion including exon 4, Chr20(GRCh38):g.36548744_36555707del]	See Moreno et al. (2016), Moreno et al. (2018)
P3 (proband in this study)	This study	NM_006097.5:c. [184+2_184+10del]; [347‐21_*594del] [compound heterozygous loss of canonical splice donor at exon 2 and deletion of exon 4, corresponding to Chr20(GRCh38):g.36545070_36545078del and Chr20(GRCh38):g.(?_36549057)_(36549844_?)del, respectively, confirmed *in trans* by parental testing]	*Gastrointestinal system*—Microcolon, intestinal hypoperistalsis, lower esophageal stricture
			*Renal system*—Fetal hydronephrosis, oligohydramnios, megacystis, bilateral hydroureteronephrosis (right kidney 11% function, left kidney 89% function), kidney stones since one year of age, requires catheterization
			*Ocular system*—Congenital bilateral mydriasis with light sensitivity
			*Cardiovascular and ventricular system* ^4^—Mild diffuse cerebral atrophy with mild ex vacuo enlargement of the ventricular system and enlarged Virchow‐Robin spaces (assessed as “diffuse microvascular disease” by neurology), ectasia of the left supraclinoid internal carotid artery, patent foramen ovale
			*Nervous system*—Progressive white matter loss, no known diagnosis, occasionally stutters or mixes up words previously well known to her, no physical manifestations
			*Respiratory syzstem*—Bronchopulmonary dysplasia (O_2_ supplementation), hospitalizations for severe respiratory infections
			*Skeletal system*—Hypermobile finger joints
			*General*—Developmental delay, heat/cold intolerance (dysregulated homeostasis?), hospitalized ~four times per year for urinary tract infections, iron deficiency
P4 (deceased)	This study	DNA not available, N/A	Autopsy findings: posterior urethral valve anomaly and enlarged bladder

a RefSeq: NC_000020.11, nucleotides 36541519‐36551447.

b GRCh38 was used; in both probands, the variants were determined to be *in trans* by parental testing.

c Adapted from (Moreno et al., 2016, 2018).

d Phenotypes in grey highlight were also observed in the previously reported family.

e To our knowledge, cerebrovascular/cardiovascular/ventricular anomalies have not yet been linked to *MYL9* dysfunction; however, numerous individuals with a related syndrome, multisystemic smooth muscle dysfunction syndrome (MSMDS) have overlapping phenotypes (Moreno et al., 2016).

Sequencing and deletion/duplication analysis of the *ACTG2* gene [the primary gene responsible for MMIHS, (Thorson et al., 2014; Wangler et al., 2014)] was performed for the proband at Prevention Genetics, and the results came back normal. The patient blood sample was sent to Fulgent Genetics to perform sequencing and deletion/duplication analysis on other genes associated with MMIHS: *LMOD1*,*MYH11*, *MYLK*, and *MYL9* (Gauthier et al., 2015; Halim, et al., 2017; Halim, et al., 2017; Moreno et al., 2018). Two heterozygous loss of function variants were observed in *MYL9*. The first was a nine base pair deletion that removes the canonical splicing donor site at exon 2 NM_006097.5:c.184+2_184+10del (confirmed by Sanger, Figure S2) and the second was an exon 4 deletion (confirmed by qPCR; Figure S3).

A similar deletion including exon 4 was observed in the heterozygous state in two individuals among a sample size of 2,504 [(Mills et al., 2011); DGV accession number esv3645738], yielding an estimated allele frequency of ~1/1,250. The observed splicing variant was also rare, only occurring in the heterozygous state in two individuals among a sample size of 125,462 in the Broad Database to yield an estimated allele frequency of 1/62,731. Parental testing confirmed that these variants were *in trans* (the proband's mother carries the exon 4 deletion and the proband's father carries the canonical splice donor deletion). Since this is only the second individual with MMIHS firmly linked to the *MYL9* gene and the gene‐disease association remains “limited” (ClinGen Gene Clinical Validity Curation Process Standard Operating Procedure, version 6), it was inappropriate to use the PVS1 ACMG evidence code (Richards et al., 2015) at any level. With the remaining evidence taken into account, each variant was assigned a technical classification of “variant of unknown significance” with the understanding that future strengthening of the gene‐disease association for *MYL9* would likely justify the reclassification each of these variants to “likely pathogenic” or “pathogenic.”

## 4 DISCUSSION

The disease association for the *MYL9* gene is not well‐established. To our knowledge, only one other report of *MYL9*‐associated MMIHS has been fully described (Moreno et al., 2018). That reported individual had a 6,964 bp homozygous deletion including part of intron 3, all of exon 4 and the 3′ UTR, and downstream intergenic sequence. Our proband also has an exon 4 deletion, though due to low upstream and downstream sequencing coverage breakpoints remain undefined. Additionally, the previously reported individual had a sibling who died at one month of age due to renal failure and other symptoms suggestive of MMIHS; however, his DNA could not be evaluated due to degradation (Moreno et al., 2016, 2018). The phenotypic landmarks of our proband, her deceased brother, and the two deceased individuals from the previous report are described in Table 1. Thus far, *MYL9*‐associated MMIHS appears to be autosomal recessive; however, it is interesting that the proband's mother had a mild but chronic gastrointestinal phenotype. Incidentally, a rare heterozygous variant (p.Glu112_Ser115del) in *MYL9* was reported in one individual with MMIHS who was negative for any changes in *ACTG2*,*MYH11* ,*MYLK*, and *LMOD1*; however, no second *MYL9* variant was found (https://spuonline.org/abstracts/2018/P25.cgi). This could suggest a possible autosomal dominant/autosomal recessive inheritance pattern, however, more clinical data are needed.

Although *MYL9 *has not been well studied in humans, in vitro functional studies demonstrate that depleting expression of murine *Myl9* by knockout of its transcriptional activator, Junb, significantly reduces wound closure and cellular contractility in mouse embryonic fibroblasts, which were restored to normal levels when either *Junb* or *Myl9* were re‐expressed by complementation (Licht et al., 2010). In human vascular smooth muscle cell (VSMC) lines, expression of MYL9 and its activator JUNB are repressed by the microRNA miR‐633 and this repression correlates with reduced proliferation and migration (Li et al., 2013). In vivo mouse experiments found that siRNA knockdown of Myl9 expression reduces invasion and lung colonization of two highly metastatic tumor cell lines, human MDA‐MB‐231 breast carcinoma and mouse B16F2 melanoma, suggesting an important role for Myl9's cytoskeletal functions in metastasis (Medjkane et al., 2009). In rats, Myl9 expression increases with age in the endothelial cells of both healthy and injured arteries, which has implications for vascular permeability and contractility (Shehadeh et al., 2011). Interestingly, Myl9 is also involved in the immune system. Myl9 protein is produced by platelets in response to inflammation and conglomerates into “Myl9 nets” in the luminal space of blood vessels, attracting CD69‐expressing lymphocytes and providing a “platform” for their migration into inflamed tissue (Kimura et al., 2017). Additionally, the importance of myosin II activity for the migratory capacity of other immune cells [e.g., dendritic cells, (Barbier et al., 2019)] suggests that loss of *MYL9* may also impair immune responses requiring cellular contraction.

Since the cellular contractility furnished by MYL9 appears to be important for many biological functions (e.g., wound closure and healing, vascular permeability, smooth muscle cell migration, metastasis, immunity) it is not surprising that our proband has a number of phenotypes apart from the classic megacystis‐microcolon‐hypoperistalsis triad. This diversity of phenotypes may arise from the ability of Myl9 to interact with a variety of myosin heavy chain II proteins in a range of different tissues and cell types, as demonstrated in mice (Park et al., 2011). Indeed, others have hypothesized that MYL9 is important for cellular contraction in tissues that utilize different actin isoforms (Moreno et al., 2018), for example, actin alpha two (*ACTA2*) which is associated with vascular disease and MSMDS (OMIM: 102620; https://www.ncbi.nlm.nih.gov/gene/59) and actin gamma two (*ACTG2*) which is associated with enteric disease and MMIHS (OMIM: 102545; https://www.ncbi.nlm.nih.gov/gene/72).

In summary, we report here the second individual with MMIHS associated with biallelic loss of function variants in the *MYL9* gene. To our knowledge, this proband is the only individual known to have survived biallelic *MYL9*‐associated MMIHS. Each genetically confirmed proband in Table 1 also had a sibling who died with symptoms suggestive of possible MMIHS, although we note that posterior urethral valve anomalies are a relatively common cause of congenital bladder obstruction in males (https://www.chop.edu/conditions‐diseases/posterior‐urethral‐valves‐puv). Additionally, since the siblings' DNA in both families could not be evaluated, the gene‐disease association remains at the “limited” level. At present, it is unclear if additional phenotypes are seen in the proband (e.g., white matter loss, kidney stones, bronchopulmonary dysplasia, etc., see Table 1) can be linked to *MYL9*, and additional genetic testing has not been performed. Finally, the clinical evidence that MYL9 is part of contractile machinery in tissues using different actin isoforms (e.g., actin alpha two vs. actin gamma two) may justify cardiovascular studies among individuals with deleterious *MYL9* variants. We suggest that *MYL9* be included on genetic testing panels ordered for MMIHS, smooth muscle myopathies, and cardiovascular phenotypes.

## CONFLICT OF INTEREST

Justin Kandler, Eleina Cox, and Yuan Xue own stock in Fulgent Genetics. Leslie Walsh declares that she has no conflicts of interest. Audrey Woerner declares that she has no conflicts of interest. Evgenia Sklirou declares that she has no conflicts of interest.

## AUTHOR CONTRIBUTIONS

JLK, YX, ES, and AW conceived and designed the study and wrote the paper that was reviewed and edited by all authors. ES, AW, LW, and EC cared for the patient and acquired the clinical data. All authors read and approved the manuscript.

## Supporting information

Fig S1‐S3
