# Supplementary material for: Compound heterozygous loss of function variants in MYL9 in a child with megacystis–microcolon–intestinal hypoperistalsis syndrome
Source: Mol Genet Genomic Med. 2020 Oct 8;8(11):e1516. doi: 10.1002/mgg3.1516 (PMC7667357; doi:10.1002/mgg3.1516)

**Supplemental Figure 1: Gene expression of *MYL9* from GTEx.** Data were retrieved from <https://gtexportal.org/home/gene/MYL9> in July, 2019. RNA expression level is reported in TPM (transcripts per million).


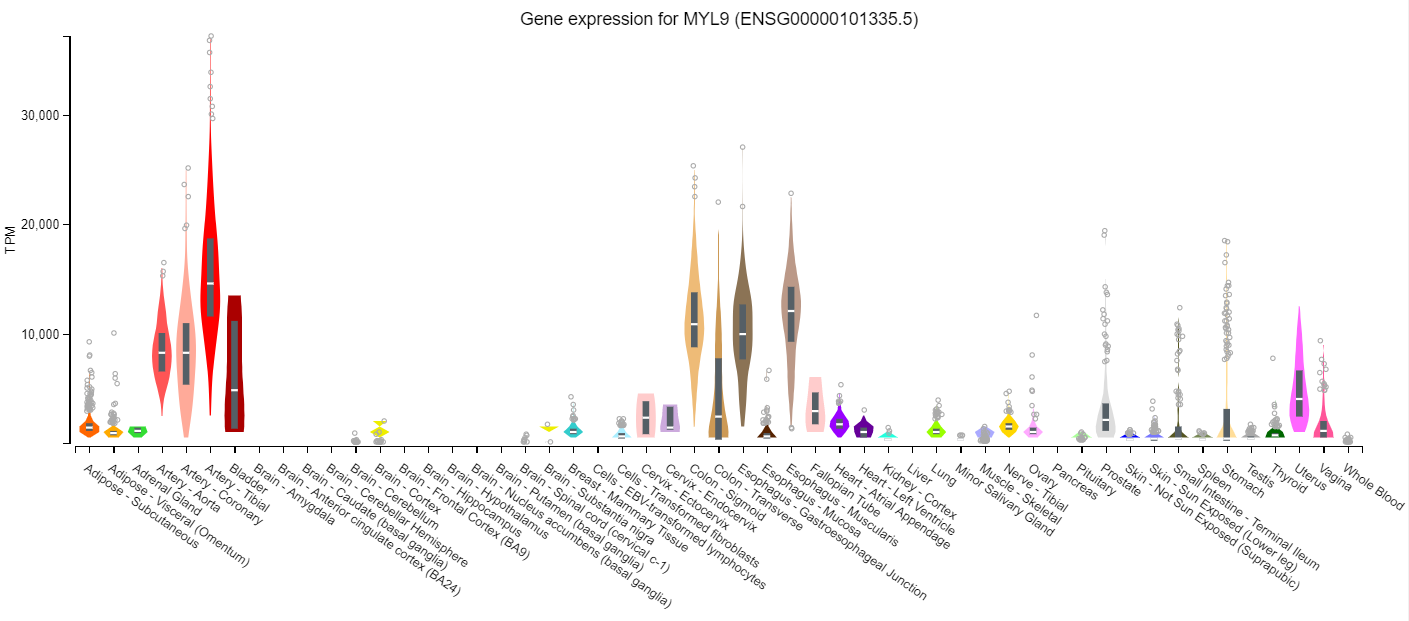


**Supplemental Figure 2: Loss of the canonical splicing donor site at exon 2 of *MYL9* in the proband.** The heterozygous NM_006097.5:c.184+2_184+10del variant is predicted by all five computational prediction tools queried within Alamut to abolish the canonical splicing donor site at the end of exon 2 of this transcript. The net effect of the deletion is to change the canonical “GU” to a “GA” in the mRNA sequence. This change is very likely to disrupt splicing of the transcript.

**
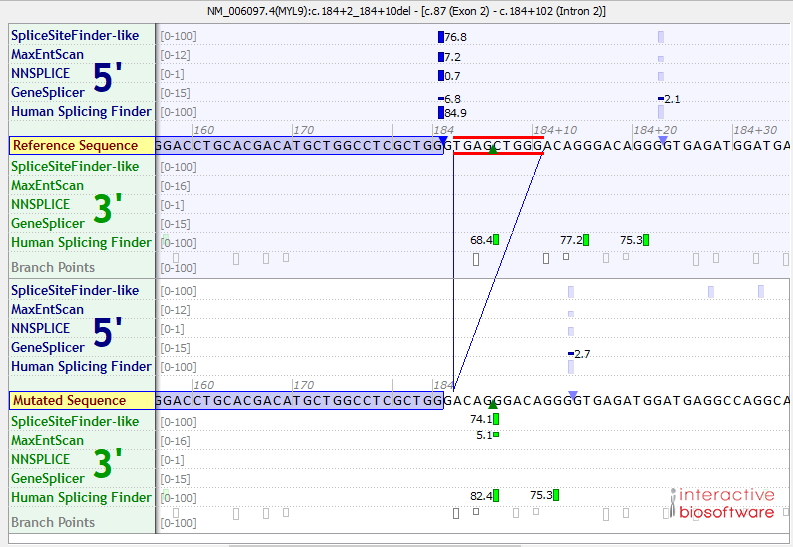
**

**Supplemental Figure 3: Illustration of the exon 4 deletion in the *MYL9* gene among individuals with *MYL9*-associated MMIHS.** Loss of exon 4 in the original proband described in Moreno et al., 2018 (breakpoints established) and in the proband reported here (breakpoints not established). Both deletions include all of the coding sequence of exon 4, in addition to the complete 3’UTR.


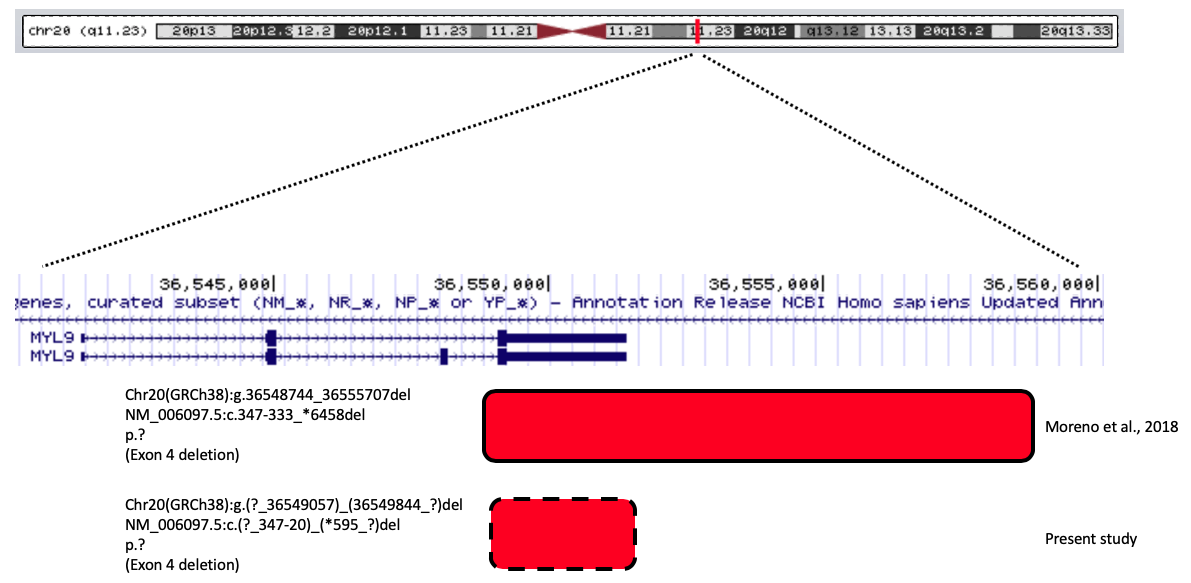

Supplement: Supplementary file 1 — Fig S1‐S3 [file MGG3-8-e1516-s001.docx]
